# Detection of high risk people for diabetes by American diabetes association risk score in PERSIAN Guilan cohort study

**DOI:** 10.1186/s12902-022-01248-4

**Published:** 2023-01-11

**Authors:** Tolou Hasandokht, Farahnaz Joukar, Saman Maroufizadeh, Zahra Sibeveih, Mohammadreza Naghipour, Zahra Hedayaztadeh, Fariborz Mansour-Ghanaei

**Affiliations:** 1 Cardiovascular Diseases Research Center, Department of Cardiology, Heshmat Hospital, School of Medicine, Guilan University of Medical Sciences, Rasht, Iran; 2 Gastrointestinal and Liver Diseases Research Center, Guilan University of Medical Sciences, Rasht, Iran; 3 Department of Biostatistics, School of health, Guilan University of Medical Sciences, Rasht, Iran

**Keywords:** Diabetes mellitus, Risk factors, Cohort studies

## Abstract

**Background:**

Diabetes mellitus (DM) is known as one of the most prevalent non communicable diseases with high cost of health services in the world. Present study was conducted to assess the frequency of high risk people for diabetes mellitus based on American Diabetes Association (ADA) risk score among Iranian people.

**Methods:**

Present study was a cross sectional study on non-diabetic subjects aged 35–70 years from 10,520 PERSIAN Guilan Cohort Study (PGCS). ADA risk score was calculated for every individual through an online calculator. Receiver operating characteristic (ROC) curves was used to assess diagnostic accuracy of the anthropometric indices to identify individuals with high risk ADA score for developing DM, represented by the area under the curve (AUC).

**Results:**

From 7989 study subjects, ADA risk score found 3874 (48.5%) and 1912 (23%) at risk for developing PreDM and DM, respectively. The results of ROC curve analyses showed the highest diagnostic value was related to waist circumference (WC) in total population and Waist to Height Ratio in both sex (0.695 total, 0.743 female, 0.744 male). The cut-points of WC in total population to identifying high risk group were 97 cm.

**Conclusions:**

A considerable number of populations were classified as high ADA risk for developing DM and PreDM that provide the importance of prevention strategies. Present study showed WC and Waist to Height Ratio have the highest diagnostic value to identify high risk people for DM.

## Introduction

Diabetes mellitus (DM) is one of the most prevalent chronic diseases in the world with high cost of medical services due to complications of the disease [1]. Over the past decade, the prevalence of diabetes has risen due to aging, urbanization, and increased unhealthy behaviors like bad dietary habits, physical inactivity [2]. According to International Federation of Diabetes (IDF) Atlas for Diabetes, Middle East region and Iran were known as one of the counties with high prevalence of diabetes. It is estimated 9.2 million Iranian individuals will have diabetes by the year 2030 [3]. Hence, this dramatic increase in the diabetes prevalence results the high economic cost for management of disease and its complications [4]. In spite of genetic susceptibility, environmental factors like unhealthy diet habits and sedentary lifestyle play an important role in the development of diabetes [5]. Evidence from studies has clearly shown early identification and behavioral intervention to lose weight, increase physical activity and choose healthy diet can significantly decrease the incidence of diabetes and prediabetes [6, 7]. To decrease the burden of DM, several guidelines and World Health Organization (WHO) recommended strategies for early detection of individuals are at risk of diabetes [8–10]. Till now, several non-invasive and easily practical risk prediction model have been developed to identifying those with high risk for diabetes including FINDRISC (Finnish Diabetes Risk Score) [11], AUSDRISK (Australian Type 2 Diabetes Risk Assessment Tool) [12], ADA (American Diabetes Association)RISK SCORE [13, 14], and a risk score had been developed in Thailand [15]. In a recent study [16] the validity of the ADA risk prediction models had been confirmed for identifying high risk individuals for type 2 diabetes in a large sample of Iranian population related to the Tehran Lipid and Glucose Study (TLGS). In the recent report, over the 70% of Guilan population were found to be overweight or obese [17] and consequently at risk of non-communicable disease. Hence, identifying high risk people and implementing community based prevention program seems to be substantial. The aim of present study was to assess the prevalence of high risk individuals for diabetes or prediabetes among Iranian individuals according to ADA risk score.

## Materials and methods

### Study design and population

This is a cross-sectional study on PGCS participants (PERSIAN Guilan Cohort Study), a prospective, population-based cohort study in Guilan has been described in detail elsewhere [17–19]. Briefly,  PGCS was conducted on 10,520 participants aged between 35 and 70 years in Guilan province, northern Iran, between October 8, 2014 and January 20, 2017 as part of the Prospective Epidemiological Research Studies in Iran (PERSIAN). Eligible subjects were contacted through phone by trained interviewers who can spoke the native language of the region to participate the study. After signed informed constant all study data including demographic characteristics, socioeconomic status, lifestyle and sleep habits, Anthropometric indices and blood pressure were recorded by trained research assistants. Also biological samples were collected. In phase 2, annually active follow up was planned for next 15 years for all participants according to the PERSIAN cohort protocol [18]. Present study data included 7989 non diabetic participants of the PGCS study. Diabetic subjects were excluded. Subjects with DM in the PGCS were defined as 1) history of diagnosed DM 2) history of anti-diabetic medication consumption 3) fasting blood sugar (FBS) > 126 in the initial cohort laboratory data [17].

### Data collection and measurement

For every participant, we retrieved data from PGCS database that were collected through interviews, physical examinations, and laboratory tests according to cohort protocol [17]. For the present study, data included demographic factors like age, sex, living location (city or rural), Marital status, Occupation (employed, unemployed), anthropometric indices including weight, height, hip and waist circumference, waist to hip ratio (WHpR) and waist to height ratio (WHtR), history of hypertension (HTN), gestational DM in women subjects and any history of DM in their first degree family like father, mother, sister or brother and finally information about physical activity. All anthropometric indices including weight, height, Hip Circumference (HC), Waist Circumference (WC), WHpR, and WHtR were measured by trained research assistants according to GCS protocol. Body mass index (BMI) was categorized as underweight (BMI < 18.5 kg/m2), normal weight (BMI = 18.5–24.99 kg/m2), overweight (BMI = 25–29.9 kg/m2) and obese (BMI ≥ 30 kg/m2). The level of physical activity was reported as metabolic equivalent rates (METs) based on self-reported daily activity PERSIAN cohort questionnaire.

The risk of developing DM or prediabetes was calculated for every individual based on ADA risk prediction model through online calculator [13] the ADA risk prediction model was developed based on American population higher than 20 years without DM to identify high risk individuals for DM or prediabetes. ADA risk score included 7 questions like age, sex, race, weight, height, family history of DM, history of gestational DM, history of HTN and physical activity. Total score was calculated between 0 and 11. The higher score represents a higher risk of diabetes. The cut point 5 or higher shows the high risk for DM and cut point 4 shows the high risk for prediabetes [13]. All required data for calculating ADA risk were extracted from cohort study. Family history of DM in ADA risk score was defined any history of diabetes in mother, father, sister or brother. Gestational diabetes in PERSIAN cohort was considered yes if women answered yes to the question “did you have a history of diabetes in pregnancy or did you have given birth a baby with ≥4 kg?” For race, all participants were defined as white. For physical activity, the question in ADA risk score tool was “are you physically active? Yes or no” Low level of physical activity in PERSIAN cohort was defined as less than mean METs rates of participants (41 METs/hour/day) that have been previously described in details [20].

### Ethics

This research project was approved by the Ethics Committee of the Gastrointestinal and Liver Disease Research Center and Guilan University of Medical Sciences (code number IR.GUMS.REC.1398.241). All participants expressed their consent for participation in the research.

#### Statistical analysis

In this study, continuous variables were expressed as mean ± standard deviation (SD) and categorical variables as frequency (percentage). One-way ANOVA and Chi-square test were used to compare demographic characteristics and anthropometric indices among normal, prediabetes, and diabetes groups. Receiver operating characteristic (ROC) curves were used to study diagnostic accuracy of the anthropometric indices for detecting patients with diabetes, represented by area under the curve (AUC). An AUC value of 0.5 indicates an entirely random classifier and an AUC value of 1 indicates perfect classifier. The best cut-off value was defined as the value with the highest accuracy that maximizes you den’s J statistic, i.e. J = sensitivity + specificity – 1. Data analysis was performed using IBM SPSS Statistics for Windows, version 26.0 (IBM Corp., Armonk, NY, USA), and a *P* < 0.05 was considered statistically significant.

## Result

### Characteristics of the participants

**Fig. 1:**
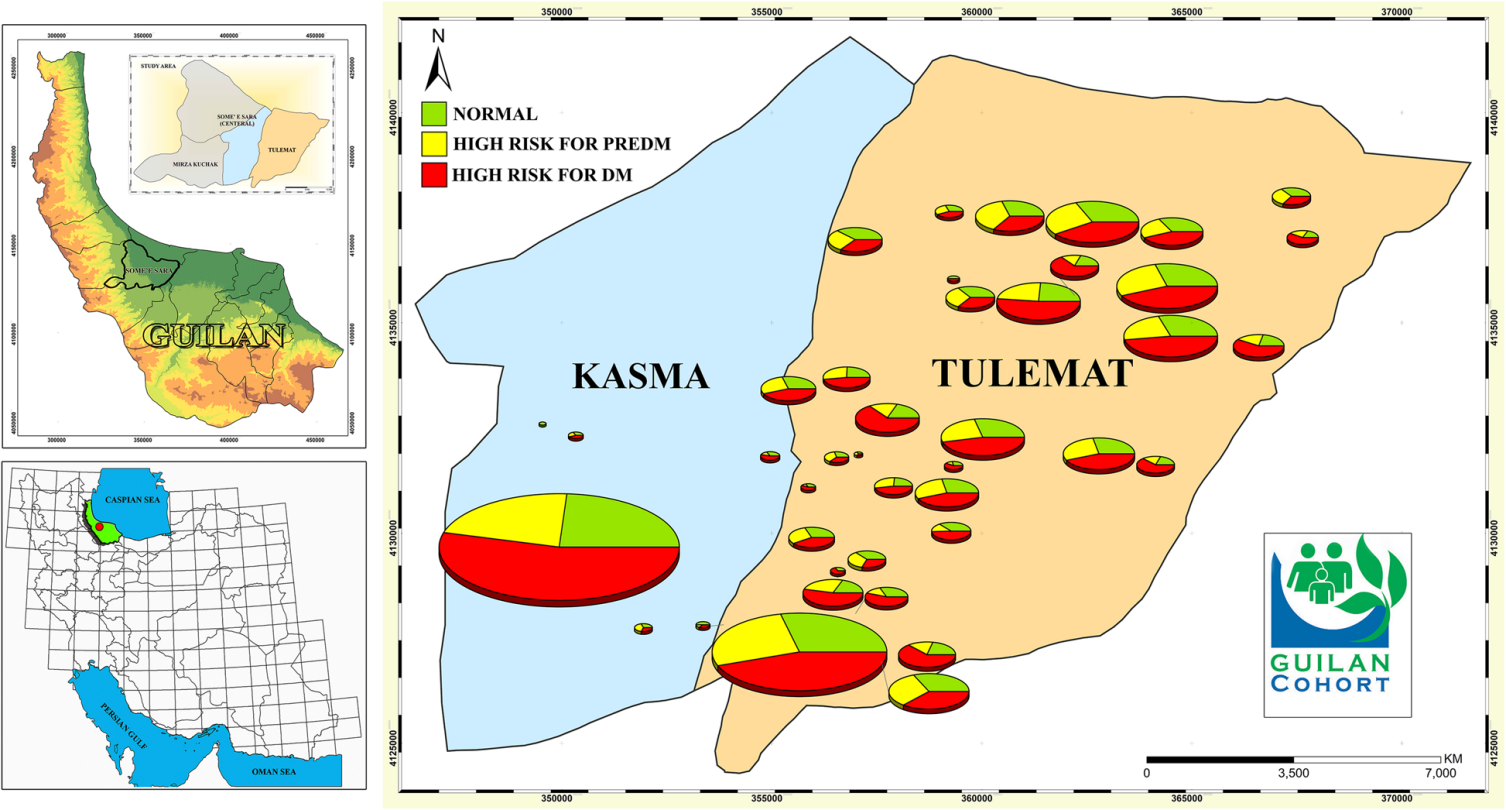
The geographic distributions of study participants according to ADA score category

Totally, of 10,520 participants, 7989 non-diabetic individuals were included in the study. Prevalence of DM in PGCS was 2531 (24.1%) [17]. Demographic characteristics and anthropometric indices of the participants are presented in Table 1. The geographic distributions of study participants according to ADA score category (normal, high risk for preDM, high risk for DM) are presented in Fig. 1. The mean age of the participants was 50.52 ± 8.75 years. More than, 51% were female, 91.2% were married, 54.9% were resident in rural areas, and 27.5% had normal BMI, 53.6% had a family history of diabetes.

**Table 1:** Demographic and clinical characteristics of adult participants based on ADA risk scores in PGSC^a^ (*n* = 7989)

	Total	Normal (N)	Prediabetes (PreD)	Diabetes (D)	*P*	
**Age**^**b**^ (y)	50.52 ± 8.75	44.62 ± 5.83	48.59 ± 7.48	54.84 ± 8.40	< 0.001	N < PreD < D
**Sex**^**c**^					< 0.001	
Male	3898 (48.8)	990 (25.4)	913 (23.4)	1995 (51.2)		
Female	4091 (51.2)	1213 (29.7)	999 (24.4)	1879 (45.9)		
**Marital status**^**c**^					< 0.001	
Single	259 (3.2)	129 (49.8)	53 (20.5)	77 (29.7)		
Married	7282 (91.2)	1969 (27.0)	1772 (24.3)	3541 (48.6)		
Widowed	348 (4.4)	65 (18.7)	68 (19.5)	215 (61.8)		
Divorced	100 (1.3)	40 (40.0)	19 (19.0)	41 (41.0)		
**Occupation**^**c**^					< 0.001	
Employed	3372 (42.2)	793 (23.5)	785 (23.3)	1794 (53.2)		
Unemployed	4617 (57.8)	1410 (30.5)	1127 (24.4)	2080 (45.1)		
**Place of residence**^**c**^					< 0.001	
Urban	3601 (45.1)	893 (24.8)	868 (24.1)	1840 (51.1)		
Rural	4388 (54.9)	1310 (29.9)	1044 (23.8)	2034 (46.4)		
**Height**^**b**^ (cm)	162.94 ± 9.35	163.38 ± 9.29	163.07 ± 9.29	162.62 ± 9.39	0.008	N > D
**Weight**^**b**^ (kg)	73.91 ± 13.54	67.49 ± 11.23	72.96 ± 12.75	78.03 ± 13.61	< 0.001	N < PreD < D
**BMI**^**c**^ (kg/m^2^)	27.90 ± 4.97	25.34 ± 4.08	27.49 ± 4.62	29.56 ± 4.94	< 0.001	N < PreD < D
Underweight	119 (1.5)	64 (53.8)	31 (26.1)	24 (20.2)	< 0.001	
Normal	2200 (27.5)	1063 (48.3)	545 (24.8)	592 (26.9)		
Overweight	3177 (39.8)	850 (26.8)	825 (26.0)	1502 (47.3)		
Obese	2493 (31.2)	226 (9.1)	511 (20.5)	1756 (70.4)		
**Family history of diabetes**^**c**^					< 0.001	N < PreD < D
No	3703 (46.4)	1435 (38.8)	904 (24.4)	1364 (36.8)		
Yes	4286 (53.6)	768 (17.9)	1008 (23.5)	2510 (58.6)		
**Physical activity**^**c**^					< 0.001	N < PreD < D
< 41 METs/hour/day	4595(57.5)	860(18.7)	1017(22.1)	2718(59.1)		
> 41 METs/hour/day	3394(42.5)	1341(39.5)	895(26.3)	1156 [22]		
**Hypertension**^**c**^					< 0.001	N < PreD < D
No	5041(63.1)	1985(39.4)	1427(28.3)	1629(32.3)		
Yes	2948(36.9)	218(7.4)	485(16.5)	2245(76.2)		
**Hip Circumference**^**b**^ (cm)	102.92 ± 9.65	99.07 ± 8.16	102.14 ± 9.07	105.50 ± 9.91	< 0.001	N < PreD < D
**Waist Circumference**^**b**^ (cm)	97.98 ± 12.32	91.64 ± 11.04	96.79 ± 11.23	102.18 ± 11.84	< 0.001	N < PreD < D
**Wrist Circumference**^**b**^ (cm)	16.69 ± 1.34	16.17 ± 1.21	16.57 ± 1.25	17.04 ± 1.34	< 0.001	N < PreD < D
**Waist/Hip Ratio**^**b**^	0.95 ± 0.06	0.92 ± 0.07	0.95 ± 0.06	0.97 ± 0.05	< 0.001	N < PreD < D
**Waist/Height Ratio**^**b**^	0.60 ± 0.09	0.56 ± 0.08	0.60 ± 0.08	0.63 ± 0.09	< 0.001	N < PreD < D

^a^ PERSIAN Guilan Cohort Study, ^b^ mean ± SD, ^c^ number (%)

### Distribution of ADA risk score

**Fig. 2:**
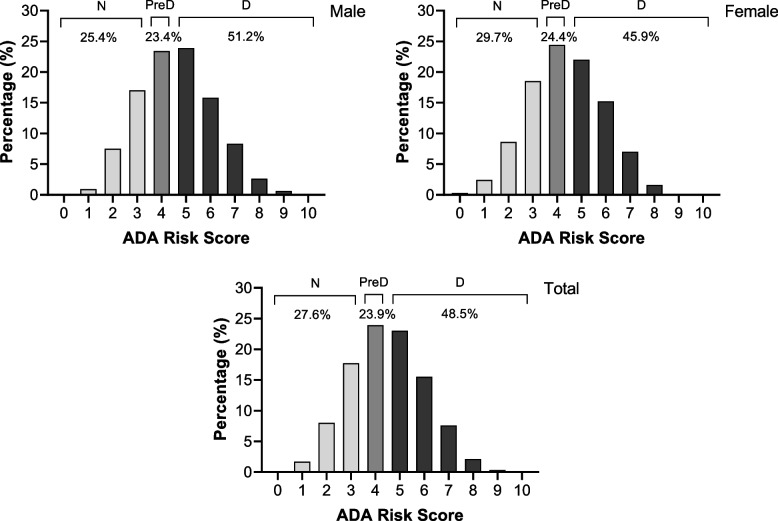
Distribution of ADA risk score among adults participants without diagnosed DM in the Persian Guilan Cohort Study (*n* = 7989). N: Normal; PreD: Prediabetes; D: Diabetes

Figure 2 presents the frequency of ADA risk scores among participants. The mean ADA risk score for all population were 4.48 (SD = 1.55), and using a recommended cut-off values, the frequency of high risk subjects for preDM and DM were, 23.9% (*n* = 1912) and 48.5% (*n* = 3874), respectively.

### Comparison of groups

As presented in Table 1, all of the anthropometric indices (i.e., BMI, HC, WC, WHpR, and WHtR) in diabetes group were higher than those in prediabetes and normal groups. In addition, all anthropometric indices in prediabetes group were also higher than in normal group.

### ROC curve analysis

Table 2 shows the cut point of anthropometric indices in the study population and also findings of the ROC curve. The results of ROC curve analyses to examine the diagnostic accuracy of the anthropometric indices for detecting patients with diabetes based on ADA risk score are presented in Table 2. Based on the AUC values, the anthropometric indices that had the highest diagnostic value was “Waist Circumference” followed by “BMI” in total population and Waist to Height Ratio according to sex in differentiating patients with diabetes and healthy subjects. Table 2.

**Table 2:** Diagnostic accuracy of anthropometric indices for detecting participants with diabetes using ROC curve analysis

	Cut-Point	Sensitivity (%)	Specificity (%)	AUC
**Male**				
Weight	76.0	54.9	68.5	0.663
BMI	24.74	77.1	56.8	0.720
Hip Circumference	100.1	54.5	69.7	0.668
Waist Circumference	93.1	65.9	66.5	0.720
Wrist Circumference	17.2	58.3	63.8	0.648
Waist to Hip Ratio	0.94	67.8	63.5	0.708
Waist to Height Ratio	0.56	59.1	76.9	0.744
**Female**				
Weight	72.5	62.5	64.1	0.676
BMI	29.44	71.3	66.6	0.724
Hip Circumference	106.4	61.8	66.5	0.683
Waist Circumference	103.1	64.3	70.3	0.728
Wrist Circumference	16.2	54.4	66.7	0.649
Waist to Hip Ratio	0.97	59.1	63.6	0.647
Waist to Height Ratio	0.64	76.1	60.8	0.743
**Total**				
Weight	73	62.1	63.0	0.670
BMI	29.16	52.8	77.2	0.693
Hip Circumference	103	55.1	67.6	0.653
Waist Circumference	97.1	66.1	63.4	0.695
Wrist Circumference	17	44.5	75.4	0.643
Waist to Hip Ratio	0.94	73.8	50.0	0.663
Waist to Height Ratio	0.59	66.1	56.8	0.666

^a^AUC: Area Under The Curve

## Discussion

Finding from PGCS showed that, near to half of non-diabetic participants (48%) were high risk for developing DM and also more than 23% were high risk for preDM. In a large survey (National Health and Nutrition Examination Survey) conducted from 1999 to 2006, ADA risk score found 35% of subjects were high risk for DM [15]. In a recent descriptive large study conducted in central of Iran, prevalence of DM and preDM was 16.1 and 24.5%, respectively [21]. Evidence shows an increase of 35% in DM prevalence in 2011 compared to 2005. In parallel with our prediction, a meta-analysis modeling study estimated 9.2 million Iranian people will have diabetes by the year 2030 [3]. This significant increase in DM prevalence and also in DM complications, implementation of prevention and control programs seems to be substantial. Finding from Iranian National Surveys (2007–2016) on 7665 and 93,733 adults with and without known diabetes showed secondary prevention in individual level was effective to control of FBS level but primary prevention in non-diabetic people had no positive effect [22].

According to our study, frequency of high risk subjects for DM were superior in male when compared to female as well as in urban area rather than rural area. Although, more subjects of PGCS population lived in rural area. In primary analysis of PGCS, diabetes was more prevalent in females (27.3%) rather than males (20.2%) [17]. The finding of PERSIAN Kharameh cohort study showed that subjects living in urban areas were more likely to display metabolic syndrome and DM than those living in rural areas [23]. Contrary to our study in Kharameh cohort study, prevalence of impaired fasting glucose in females was higher than males. On the other hand, according to International Diabetes Federation, there were about 14 million more men than women with diabetes (198 million men vs 184 million women) in 2013 and it seems the difference increases to 15 million (303 million men vs 288 million women) by 2035 [24]. Furthermore, in a cross sectional study among adults aged 20–80 years in northern part of Iran was observed DM were most prevalent in males than females [25].

In present study, subjects with high risk for DM had higher waist and hip circumference, waist/hip ratio and waist/height ratio compared to those with low risk group and also to preDM group. Correlation of obesity and risk of developing DM was reported in previous evidence [25–28].

Our study showed that, the calculated cut points of BMI, Hip Circumference and Waist Circumference in women were higher than in men. Parallel to the findings of present study, PERSIAN study on 17 cohort centers showed the prevalence of overweight and obesity in male and female was 60 and 78%, respectively [29]. Also, based on another cross sectional study on the PERSIAN Guilan Cohort Study, 86% of women and 68% of men were in BMI > 25 category. As well as, the frequency of women and men with high waist circumference was 93 and 21%, respectively [30].

According to our finding, WC followed by BMI had the highest diagnostic value in identifying high risk men and women for developing DM. Although, a prospective study on Iranian adult men in 2006 indicated WHtR is better than BMI and WC in detecting urban men population who was at risk of diabetes [31]. On the other hand, according to a study based on Isfahan Cohort Study (ICS), WC compared to other anthropometric indices was better indicator of metabolic syndrome in Iranian women and men population [32]. Furthermore, previous researches showed WC is strongly related to all-cause and cardiovascular mortality with or without adjustment for BMI [33, 34]. Recent review indicated waist circumference is associated with health outcomes within all BMI categories in every sex and age [35].

In the present study, cut-points of WC and BMI for identifying high risk people for developing DM were 97 and 29, respectively which were higher than the recommended cutoff for major CVD risk factors in previous studies [32, 36, 37]. The first Iranian study on anthropometric indices proposed WC and BMI cut-offs for detecting DM, between 82 and 95 cm and 25–29 in women and men, in various age groups [36]. Iranian National Committee of Obesity reported people with WC of ≥90 cm are at high risk for CVD event [37].

Cut-points of WHpR to identify high risk individual for developing DM in the present study were 0.94 that somewhat close to the recommended cut point in other studies. For example, P Mirmiran et al. found cut-points of WHR between 0.86 and 0.97 for men and between 0.78 and 0.92 for women were high risk for various CVD risk factors [36]. In our study, the diagnostic values of WHpR and WHtR in identifying high risk people for DM were relatively similar. The diagnostic value of WHtR in Chinese cohort study was reported 0.679 that was in parallel with our finding(AUC: 0.666) [38]. A recent study in middle east region showed WHtR can better predict the risk of DM and also HTN [39]. Finding from a population based study of 1852 Iranian males aged ≥20 years showed WHtR was a strong predictor for developing type 2 diabetes in the future [31].

In total, finding high risk people in individual and community level may help people and policy makers to develop and plan prevention strategies. We detect considerable numbers of Iranian adult lived in northern part of Iran were high risk for developing DM and PreDM. Hence, note to lifestyle modification in individual and community level seems to be substantial. Our study was based on PGCS with large sample size and accurate data collection. Data collection and measurement were based on Persian cohort study standards that increase the precision of the finding. However, this study involves some limitation. Firs due to cross sectional nature of present study design, we couldn’t define the actual risk of study subjects and compare to their calculated risk. However, the validity and sensitivity of ADA risk score among PGCS population could be assess in the future years using long term follow up duration. Furthermore, some study variables like physical activity, history of gestational diabetes were measured based on self-reported that increases the probability of recall bias.

## Conclusion

In conclusion, present study showed considerable number of people lived in the northern part of Iran were classified in high risk category of ADA risk score for developing DM and PreDM. High risk group were more prevalent in male rather than female and also urban residents. According to our finding, waist circumference and Waist to Height Ratio appears to be better diagnostic value in identifying high risk people for developing DM.

## Data Availability

Datasets used during the study are available from the corresponding author on reasonable request.

## Abbreviations

DM: Diabetes,Mellitus
ADA: American Diabetes Association
PGCS: PERSIAN Guilan Cohort Study
ROC: Receiver operating characteristic
AUC: Area under the Curve
WC: Waist Circumference
WHO: World Health Organization
TLGS: Tehran Lipid and Glucose Study
GCS: Guilan cohort study
WHpR: Waist to Hip Ratio
WHtR: Waist to Height Ratio
HTN: History of Hypertension
HC: Hip Circumference
METs: Metabolic Equivalent Rates
BMI: Body Mass Index

## References

[1] de Lapertosa SG, de Moura AF, Decroux CH, Duke L, Hammond L, Jacobs E, et al. IDF Diabetes Atlas 9th edition. 2019. Available from: https://diabetesatlas.org/atlas/ninth-edition/.

[2] Haghdoost A, Rezazadeh-Kermani M, Sadghirad B, Baradaran H (2009). Prevalence of type 2 diabetes in the Islamic Republic of Iran: systematic review and meta-analysis.

[3] Javanbakht M, Mashayekhi A, Baradaran HR, Haghdoost A, Afshin A (2015). Projection of diabetes population size and associated economic burden through 2030 in Iran: evidence from micro-simulation Markov model and Bayesian meta-analysis. PLoS One.

[4] Esteghamati A, Larijani B, Aghajani MH, Ghaemi F, Kermanchi J, Shahrami A (2017). Diabetes in Iran: prospective analysis from first nationwide diabetes report of National Program for prevention and control of diabetes (NPPCD-2016). Sci Rep.

[5] Said MA, Verweij N, van der Harst P (2018). Associations of combined genetic and lifestyle risks with incident cardiovascular disease and diabetes in the UK biobank study. JAMA Cardiol.

[6] Gong Q, Zhang P, Wang J, Ma J, An Y, Chen Y (2019). Morbidity and mortality after lifestyle intervention for people with impaired glucose tolerance: 30-year results of the Da Qing diabetes prevention outcome study. The Lancet Diabetes & Endocrinol.

[7] Galaviz KI, Weber MB, Straus A, Haw JS, Narayan KMV, Ali MK (2018). Global diabetes prevention interventions: a systematic review and network meta-analysis of the real-world impact on incidence, weight, and glucose. Diabetes Care.

[8] Organization WH (2013). Global action plan for the prevention and control of noncommunicable diseases.

[9] Alberti KGMM, Zimmet P, Shaw J (2007). International diabetes federation: a consensus on type 2 diabetes prevention. Diabet Med.

[10] Abbasi A, Bakker SJ, Corpeleijn E, van der A DL, Gansevoort RT, Gans RO, et al. Liver function tests and risk prediction of incident type 2 diabetes: evaluation in two independent cohorts. PloS one. 2012;7(12):e51496. 10.1371/journal.pone.0051496 PMC3524238 23284703

[11] Lindstrom J, Tuomilehto J (2003). The diabetes risk score: a practical score to predict risk of type two diabetes. Diabetes Care.

[12] Chen L, Magliano DJ, Balkau B, Colagiuri S, Zimmet PZ, Tonkin AM (2010). AUSDRISK: an Australian type 2 diabetes risk assessment tool based on demographic, lifestyle and simple anthropometric measures. Med J Aust.

[13] American Diabetes Association. 2007. Available from: https://diabetes.org/diabetes/risk-test.

[14] Bang H, Edwards AM, Bomback AS, Ballantyne CM, Brillon D, Callahan MA, et al. Development and validation of a patient self-assessment score for diabetes risk. Annals of internal medicine. 2009;151(11):775-83. 10.1059/0003-4819-151-11-200912010-00005 PMC3633111 19949143

[15] Aekplakorn W, Bunnag P, Woodward M, Sritara P, Cheepudomwit S, Yamwong S (2006). A risk score for predicting incident diabetes in the Thai population. Diabetes Care.

[16] Lotfaliany M, Hadaegh F, Asgari S, Mansournia MA, Azizi F, Oldenburg B (2019). Non-invasive risk prediction models in identifying undiagnosed type 2 diabetes or predicting future incident cases in the Iranian population. Arch of Iran med.

[17] Mansour-Ghanaei F, Joukar F, Naghipour MR, Sepanlou SG, Poustchi H, Mojtahedi K (2019). The PERSIAN Guilan cohort study (PGCS). Arch of Iran med.

[18] Poustchi H, Eghtesad S, Kamangar F, Etemadi A, Keshtkar AA, Hekmatdoost A, et al. Prospective Epidemiological Research Studies in Iran (the PERSIAN Cohort Study): Rationale, Objectives, and Design. Am J Epidemiol. 2018;187(4):647–55. 10.1093/aje/kwx314 PMC6279089 29145581

[19] Eghtesad S, Mohammadi Z, Shayanrad A, Faramarzi E, Joukar F, Hamzeh B (2017). The PERSIAN cohort: providing the evidence needed for healthcare reform. Arch of Iran med.

[20] Kazemi Karyani A, Karmi Matin B, Soltani S, Rezaei S, Soofi M, Salimi Y, et al. Socioeconomic gradient in physical activity: findings from the PERSIAN cohort study. BMC public health. 2019;19(1):1312. 10.1186/s12889-019-7715-z PMC6802340 31638932

[21] Mirzaei M, Rahmaninan M, Mirzaei M, Nadjarzadeh A (2020). Epidemiology of diabetes mellitus, pre-diabetes, undiagnosed and uncontrolled diabetes in Central Iran: results from Yazd health study. BMC Public Health.

[22] Malekzadeh H, Lotfaliany M, Ostovar A, Hadaegh F, Azizi F, Yoosefi M (2020). Trends in cardiovascular risk factors in diabetic patients in comparison to general population in Iran: findings from National Surveys 2007–2016. Sci Rep.

[23] Nikbakht H-A, Rezaianzadeh A, Seif M, Ghaem H (2020). Prevalence of metabolic syndrome and its components among a population-based study in south of Iran, PERSIAN Kharameh cohort study. Clin Epidemiol and Glob Health.

[24] Atlas ID. Brussels, Belgium: international diabetes federation; 2013. International Diabetes Federation (IDF). 2017;147.

[25] Hajian-Tilaki K, Heidari B (2015). Is waist circumference a better predictor of diabetes than body mass index or waist-to-height ratio in Iranian adults?. Int J Prev Med.

[26] Hadaegh F, Bozorgmanesh MR, Ghasemi A, Harati H, Saadat N, Azizi F (2008). High prevalence of undiagnosed diabetes and abnormal glucose tolerance in the Iranian urban population: Tehran lipid and glucose study. BMC Public Health.

[27] Esteghamati A, Etemad K, Koohpayehzadeh J, Abbasi M, Meysamie A, Noshad S (2014). Trends in the prevalence of diabetes and impaired fasting glucose in association with obesity in Iran: 2005–2011. Diabetes Res Clin Pract.

[28] Shabnam A-A, Homa K, Reza M-TM, Bagher L, Hossein FM, Hamidreza A (2012). Cut-off points of waist circumference and body mass index for detecting diabetes, hypercholesterolemia and hypertension according to National non-Communicable Disease Risk Factors Surveillance in Iran. Arch of med scie: AMS.

[29] Najafi F, Soltani S, Karami Matin B, Kazemi Karyani A, Rezaei S, Soofi M (2020). Socioeconomic-related inequalities in overweight and obesity: findings from the PERSIAN cohort study. BMC Public Health.

[30] Mansour-Ghanaei R, Mansour-Ghanaei F, Naghipour M, Joukar F, Atrkar-Roushan Z, Tabatabaii M (2018). The role of anthropometric indices in the prediction of non-alcoholic fatty liver disease in the PERSIAN Guilan cohort study (PGCS). J of Med and Life.

[31] Hadaegh F, Zabetian A, Harati H, Azizi F (2006). Waist/height ratio as a better predictor of type 2 diabetes compared to body mass index in Tehranian adult men-a 3.6-year prospective study. Exp Clin Endocrinol Diabetes.

[32] Gharipour M, Sarrafzadegan N, Sadeghi M, Andalib E, Talaie M, Shafie D, et al. Predictors of metabolic syndrome in the Iranian population: waist circumference, body mass index, or waist to hip ratio? Cholesterol. 2013;2013. 10.1155/2013/198384 PMC3619538 23634297

[33] Cerhan JR, Moore SC, Jacobs EJ, Kitahara CM, Rosenberg PS, Adami H-O, et al., editors. A pooled analysis of waist circumference and mortality in 650,000 adults. Mayo Clinic Proceedings; 2014: Elsevier. 10.1016/j.mayocp.2013.11.011 PMC4104704 24582192

[34] Song X, Jousilahti P, Stehouwer C, Söderberg S, Onat A, Laatikainen T (2013). Comparison of various surrogate obesity indicators as predictors of cardiovascular mortality in four European populations. Eur J Clin Nutr.

[35] Ross R, Neeland IJ, Yamashita S, Shai I, Seidell J, Magni P (2020). Waist circumference as a vital sign in clinical practice: a consensus statement from the IAS and ICCR working group on visceral obesity. Nat Rev Endocrinol.

[36] Mirmiran P, Esmaillzadeh A, Azizi F (2004). Detection of cardiovascular risk factors by anthropometric measures in Tehranian adults: receiver operating characteristic (ROC) curve analysis. Eur J Clin Nutr.

[37] Fereidoun Azizi M, Davood Khalili M, Hassan Aghajani M, Alireza Esteghamati M, Farhad Hosseinpanah M, Alireza Delavari M (2010). Appropriate waist circumference cut-off points among Iranian adults: the first report of the Iranian National Committee of obesity. Arch Iran Med.

[38] Ding J, Chen X, Bao K, Yang J, Liu N, Huang W (2020). Assessing different anthropometric indices and their optimal cutoffs for prediction of type 2 diabetes and impaired fasting glucose in Asians: the Jinchang cohort study. J of Diabetes.

[39] Khader Y, Batieha A, Jaddou H, El-Khateeb M, Ajlouni K (2019). The performance of anthropometric measures to predict diabetes mellitus and hypertension among adults in Jordan. BMC Public Health.

